# Two-hybrid analysis of Ty3 capsid subdomain interactions

**DOI:** 10.1186/1759-8753-1-14

**Published:** 2010-05-05

**Authors:** Min Zhang, Liza SZ Larsen, Becky Irwin, Virginia Bilanchone, Suzanne Sandmeyer

**Affiliations:** 1 Department of Biological Chemistry, University of California, Irvine, CA, USA; 2 Department of Microbiology and Molecular Genetics, University of California, Irvine, CA, USA; 3 Institute for Genomics and Bioinformatics, University of California, Irvine, CA, USA

## Abstract

**Background:**

The yeast retrotransposon Ty3 forms stable virus-like particles. Gag3, the major structural protein, is composed of capsid, spacer and nucleocapsid domains. The capsid domain of Gag3 was previously modeled as a structure similar to retrovirus capsid.

**Findings:**

Two-hybrid analysis was used to understand the interactions that contribute to particle assembly. Gag3 interacted with itself as predicted based on its role as the major structural protein. The N-terminal subdomain (NTD) of the capsid was able to interact with itself and with the C-terminal subdomain (CTD) of the capsid, but interacted less well with intact Gag3. Mutations previously shown to block particle assembly disrupted Gag3 interactions more than subdomain interactions.

**Conclusions:**

The findings that the NTD interacts with itself and with the CTD are consistent with previous modeling and a role similar to that of the capsid in retrovirus particle structure. These results are consistent with a model in which the Gag3-Gag3 interactions that initiate assembly differ from the subdomain interactions that potentially underlie particle stability.

## Findings

The Ty3 retrotransposon in budding yeast forms virus-like particles (VLPs) comprised of precursor Gag3 and Gag3-Pol3 polyproteins [1,2]. Previous alanine-scanning mutagenesis indicated that the N-terminal domain (NTD) of the structural polyprotein Gag3 plays an important role in VLP formation [3]. During maturation, Gag3 is processed into 24 kDa capsid (CA), 27 kDa CA-spacer (SP), 3 kDa SP, and 7 kDa nucleocapsid (NC) protein by the Ty3 protease. Unlike most retrovirus cores, these cytoplasmic particles remain stable after proteolytic maturation.

**Figure 1:**
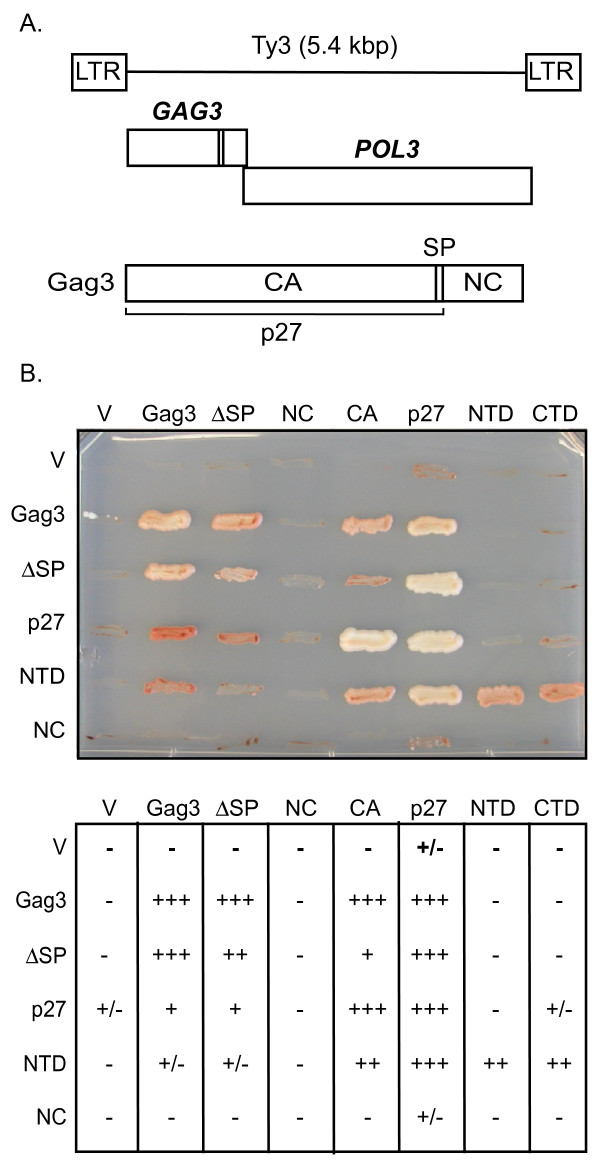
**Interaction of Ty3 Gag3 subdomains shown by interactions between Gal4 DNA binding domain (BD) and activation domain (AD) fusions to Ty3 Gag3 subdomains**. Interaction restores Gal4 mediated activation of *ADE2 *under control of the *GAL1-10 *upstream activating sequences (UASs) and growth on medium lacking adenine. Cells expressing *ADE2 *have reduced accumulation of a red adenine biosynthetic intermediate. Yeast strain yAH109 was transformed with two-hybrid pGAD or pGBK vectors (V) or expression plasmids. Streaks are representative of independent transformants growing on synthetic dextrose medium lacking tryptophan, leucine, and adenine. Gal4-BD fusions are labeled in the top row and shown in columns. Gal4-AD fusions are labeled in the left column and shown in rows. Scoring is based on four individual transformants. The most variability was observed among individual transformants expressing Gag3 and capsid (CA)-N-terminal domain fusions.

Two-hybrid analysis [4] was used to better understand the contributions of Gag3 subdomains to formation and stability of the Ty3 VLP. Fusions of Gag3 and derivatives to the C-terminus of the Gal4-BD tagged with c-Myc were expressed from the high-copy, *TRP1*-marked pGBK vector (Clontech, Palo Alto, CA, USA). Fusions of Gag3 and derivatives to the C-terminus of the Gal4-AD tagged with HA were expressed from the *LEU2*-marked high-copy plasmid pGAD T7 (pGAD). These fusions were constructed by amplifying the appropriate regions from Ty3 Gag3 subclones in pGEM (Invitrogen, Carlsbad, CA, USA) using polymerase chain reaction (PCR) primers containing *Nde*I and *Bam*HI sites at the 5' and 3' outside ends, respectively and ligating fragments to the pGBK and pGAD vectors linearized with *Nde*I and *Bam*HI. Constructs (Table 1) were confirmed by DNA sequence analysis (GeneWiz, South Plainfield, NJ, USA). Fusion proteins in these vectors are expressed under the constitutive *ADH1 *promoter. Two-hybrid plasmids and negative control vector plasmids were transformed into yeast strain yAH109 (*MATa trp1-901 leu2-3*, *112 ura3-52 his3-200 gal4Δ gal80Δ LYS2::GAL1*_*UAS/TATA*_*HIS3 GAL2*_*UAS/TATA*_-*ADE2, URA3::MEL1*_*UAS/TATA*_-*lacZ*), which has *ADE*2, *HIS*3, *Lac*Z, and *MEL1 *reporters regulated by the *GAL4 *responsive upstream activating sequences (UASs) (Clontech). Preliminary tests showed that expression of *ADE2*, which results in cream-colored colonies and growth in medium lacking adenine, provided the most reliable detection of two-hybrid interaction (data not shown). The yAH109 transformants containing pairwise combinations of pGAD and pGBK plasmids were selected on medium lacking tryptophan and leucine. Four isolates from each transformation were restreaked and then replica plated onto complete synthetic medium lacking adenine, tryptophan and leucine and grown at 30°C for evaluation of color development and growth. We first tested Gag3, CA, p27, and NC fusions in both vectors in all combinations. A mutant deleted for SP residues 208 to 232 (ΔSP) was also assayed (K Christiansen, MZ, VB and SBS, unpublished results). AD-CA transformants were not recovered as stable colony isolates in repeated attempts. Other constructs were shown to be positive for expression of the appropriate fusion by immunoblot analysis using rabbit polyclonal antibodies against Gal4 AD and Gal4 BD (Upstate Biotechnology, Lake Placid, NY, USA) or CA [5] (data not shown) [6]. With the exception of p27, which produced a slight amount of background growth in cells with both vectors, these domains were negative for reporter activation when expressed from either vector in the presence of the other vector (Figure 1, Additional files 1, 2, 3, 4, 5, 6, 7, 8, 9, 10, 11, 12).

**Table 1:** Two-hybrid plasmids

Vector/plasmid	*Nde*I/*Bam*HI insert	Gag3 residues	Mutation(s)
pGBK T7 (with Gal4-BD):			
pMZ2667	Gag3	1-290	WT
pMZ2668	Gag3 ΔSP	Δ207-233	WT
pMZ2669	p27	1-233	WT
pMZ2670	CA	1-207	WT
pMZ2671	CA-NTD	1-135	WT
pMZ2672	CA-CTD	136-207	WT
pMZ2673	NC	234-290	WT
pMZ2676	Gag3	1-290	D60A/R63A (M4)
pMZ2652	Gag3	1-290	E148A/K149A (M13)
pMZ2688	Gag3	1-290	E190A/R191A (M18)
pMZ2650	Gag3	1-290	G87A (MHR2)
pMZ2651	Gag3	1-290	F93A (MHR4)
pVB2832	CA-CTD	136-207	E148A/K149A (M13)
pVB2833	CA-CTD	136-207	E190A/R191A (M18)
pGAD T7 (with Gal4-AD):			
pMZ2677	Gag3	1-290	WT
pMZ2678	Gag3 ΔSP	Δ207-233	WT
pMZ2679	p27	1-233	WT
pMZ2680	CA	1-207	WT
pMZ2681	CA-NTD	1-135	WT
pMZ2682	CA-CTD	136-207	WT
pMZ2683	NC	234-290	WT
pMZ2686	Gag3	1-290	D60A/R63A (M4)
pMZ2655	Gag3	1-290	E148A/K149A (M13)
pMZ2689	Gag3	1-290	E190A/R191A (M18)
pMZ2653	Gag3	1-290	G87A (MHR2)
pMZ2654	Gag3	1-290	F93A (MHR4)
pVB2829	CA-NTD	1-135	D60A/R63A (M4)
pVB2830	CA-NTD	1-135	G87A (MHR2)
pVB2831	CA-NTD	1-135	F93A (MHR4)

AD = activation domain; BD = binding domain; CA = capsid; CTD = C-terminal domain; NC = nucleocapsid; NTD = N-terminal domain; SP = spacer; WT = wild type.

Homotypical interactions (except for CA) and heterotypical interactions were assayed (Figure 1). Gag3, ΔSP, and p27 interacted in both vector contexts and in all combinations tested. BD-CA interacted with AD-Gag3, AD-ΔSP, and AD-p27. NC fusions were expressed (data not shown), but failed to interact with any other fusion (Figure 1). Together these results indicated that the CA domain is capable of interactions initiated in the absence of SP and NC domains. The strong interactions of p27 with itself, compared to homotypical interactions of Gag3 or ΔSP suggested that the NC domain makes a slight negative contribution.

Among the previously described alanine-scanning mutations in Ty3 Gag3 [3] were ones that appeared to disrupt particle morphogenesis at specific stages or in specific ways. We reasoned that the interactions disrupted in particular mutants could be inferred from the effects of mutations in two-hybrid interactions. The Ty3 CA NTD from amino acids 86 to 100 includes a motif similar to the retroviral major homology region (MHR)(QGX_2_EX_2_FX_2_FX_3_L) [7-9]. Alanine substitutions at the second and fourth conserved positions in this motif (MHR2/G87A and MHR4/F93A) reduced VLP formation and processing [3]. Within the CA domain of Gag3, two sets of mutations, D60A/R63A and E148A/K149A, resulted in small Ty3 protein clusters, disrupted P-body component association, and blocked assembly and processing. One pair of alanine substitutions in the CA C-terminal domain (CTD), E190A/R191A, caused extensive cytoplasmic Ty3 protein filament formation, but did not disrupt association with P-body components. These mutations were introduced into the Gag3 two-hybrid system. Constructs were evaluated in all possible combinations with each other and with wild-type Gag3 (Table 2,, Additional files 1, 2, 3, 4, 5, 6). Gag3 fusions containing mutations that blocked assembly failed to show homotypical interactions and heterotypic interactions among themselves. Homotypical interactions of E190A/R191A Gag3, which was associated with filament formation, were at least as strong as those of wild-type Gag3. Wild-type binding domain (BD)-Gag3 or BD-E190A/R191A showed heterotypical interactions with activation domain (AD)-D60A/R63A, AD-MHR2, AD-MHR4, and AD-E148A/K149A. These results suggested that the assembly mutations act by disrupting intermolecular interfaces rather than by causing global misfolding of the Gag3 molecule.

**Table 2:** Interactions of Gag3 mutants

	V	Gag3	D60A/R63A	MHR2	MHR4	E148A/K149A	E190A/R191A
V	-	-	-	-	-	-	-

Gag3	-	+++	-	-	-	-	+++

D60A/R63A	-	+	-	-	-	-	++

MHR2	ND	++	-	-	-	-	++

MHR4	ND	++	-	-	-	-	++

E148A/K149A	-	++	-	-	-	-	++

E190A/R191A	-	+++	-	-	-	-	+++

Top row, Gal4-BD fusions; first column, Gal4-AD fusions; V, first row = pGBK vector; V, first column = pGAD vector; AD = activation domain; BD = binding domain; CTD = C-terminal domain; NTD = N-terminal domain; ND = not determined.

For images, see Additional files 1, 2, 3, 4, 5, 6.

Studies of retroviral CA assemblies show a network of CA NTD hexamers connected via CA CTD interactions (reviewed in [10]). These interactions are elucidated in more detail by studies of the crystal structures of the Moloney murine leukemia virus NTD hexamer [11] and full-length HIV1 CA [12,13]. Ty3 CA is also predicted to have CA-NTD and CA-CTD alpha helical bundles [3] and capsomeres with sixfold symmetry have been imaged on the surface of Ty3 immature and mature VLPs by atomic force microscopy, suggesting that CA NTD forms a surface hexameric network analogous to what occurs in mature retrovirus cores [2]. In order to test for the independent contributions of the NTD and CTD to interactions occurring during VLP assembly, these domains were expressed in the two-hybrid vectors. Expression was confirmed for AD-NTD and BD-NTD, and BD-CTD. AD-CTD transformants, similar to AD-CA transformants, could not be isolated, and it was concluded that expression of this fusion was toxic. Interactions were tested between NTD and CTD and between each of them and Gag3, CA, p27, ΔSP and NC (Figure 1 and Additional file 7). Interactions between AD-NTD and BD-NTD and between AD-NTD and BD-CTD were readily detected. Interactions were marginal and variable between NTD and Gag3 and ΔSP, but were readily detected between AD-NTD and BD-p27 and BD-CA. These results showed that the isolated NTD and CTD interact and that the target of the interaction, in the case of the NTD, is less accessible in the precursor Gag3 and ΔSP than in processing products p27 and CA.

In order to test whether mutations in Gag3 that affected assembly and homotypical and heterotypical interactions of intact Gag3 changed the exposure of NTD and CTD subdomains and therefore their ability to interact, interaction of these subdomains with mutant Gag3 was examined (Table 3, Additional files 8 and 9). No mutation in AD-Gag3 enhanced the ability of Gag3 to interact with BD-NTD or allowed it to interact with BD-CTD, suggesting that these mutations did not disrupt assembly by causing premature exposure of subdomain interaction sites. In order to determine which, if any, of the subdomain interactions might be directly affected by these mutations, the mutations were also introduced into the subdomains. Because AD-CTD was toxic and the BD-NTD reacted with fewer partners than AD-NTD, the mutations were introduced into BD-CTD and AD-NTD. Introduction of assembly mutations MHR2 and MHR4 into the NTD slightly weakened Gag3 AD-NTD interaction (Tables 3, 4, Additional file 10), and introduction of D60A/R63A further diminished the interaction. The effect of mutations on NTD-NTD and NTD-CTD interactions were also evaluated (Table 4, Additional files 11 and 12). Although the D60A/R63A mutation had a negative effect on NTD interactions, surprisingly, MHR2 and MHR4 mutations slightly improved interactions between NTD domains. All three mutations in the AD-NTD context enhanced interactions with the CTD. Introduction of E148A/K149A or E190A/R191A mutations into BD-CTD also did not significantly affect interaction with wild-type or mutant NTDs. Thus, overall the effect of mutations that disrupt assembly was most noticeable at the level of Gag3 polyprotein interactions. The E190A/R191A mutation, which occurs close to the end of the mature CA and causes filamentation [3], had no effect or slightly increased interaction. The evidence that interaction is not disrupted, coupled with the highly-ordered mutant structure suggests that the mutation could have a kinetic effect on assembly, thereby trapping Gag3 in an aberrant multimeric form.

**Table 3:** Effects of mutations in Gag3 on NTD and CTD interactions

	V	Gag3	NTD	CTD
V	-	-	-	-

Gag3	-	+++	-	-

NTD	-	+/-	++	++

D60A/R63A	-	+	-	-

MHR2	-	++	-	-

MHR4	-	++	-	-

E148A/K149A	-	++	-	-

E190A/R191A	-	+++	-	-

For images, see Additional files 7, 8, 9.

Abbreviations as in Table 2

**Table 4:** Effects of mutations on NTD and CTD interactions

	V	Gag3	NTD	CTD	CTD E148A/K149A	CTD E190A/R191A
V	-	-	-	-	-	-

Gag3	-	+++	-	-	-	-

NTD	-	+/-	++	++	++	++

NTD D60A/R63A	-	-	+	+++	++	++

NTD MHR2	-	+/-	+++	+++	+++	+++

NTD MHR4	-	+/-	+++	+++	+++	+++

For images, see Additional files 10, 11, 12.

Abbreviations as in Table 2

Two-hybrid analysis readily detected interactions between Gag3 polyproteins. Nevertheless there were asymmetries in the interactions introduced by the expression context. These included apparent toxicity of AD-CA and AD-CTD, detection of stronger interactions of BD-P27 compared to AD-P27, and of AD-NTD compared to BD-NTD (Figure 1). This could be explained by context-dependent exposure of interaction domains or even by context effects on nuclear entry. Thus, the positive interactions coupled with the effects of mutations or deletions on those interactions rather than the lack of interaction in one or the other expression context offer the most meaningful outcomes of our study. Two-hybrid assays showed that the CA domain interacts independent of the NC domain. This is in contrast to the apparent situation with some retroviruses in which interactions are dependent upon NC subdomains [14-16]. We speculate that this interaction contributes to the distinctive stability of the Ty3 particle. Examination of ΔSP, p27 and CA showed that interactions comparable to those between Gag3 polyproteins occur in the absence of SP and NC. This is consistent with other studies that show that defective Ty3 multimers form in the absence of these domains [17,18] (SBS and K Christiansen, University of CA, Irvine, unpublished results).

Recent 3D structures of full-length CA molecules in assembled HIV1 particles viewed as 2D crystals by cryoelectron microscopy (cryoEM) underscore three types of interactions with retroviral CA assembled into hexameric lattice structures: NTD-NTD and CTD-NTD intermolecular interactions and CTD-CTD interhexameric interactions [12,19]. NTD-NTD interactions and NTD-CTD interactions are also supported by EM studies of *in vitro *assembly intermediates of RSV [20]. Based on the similar 3D structures of different retroviral CA proteins, these interactions are believed to be generally conserved among retroviruses. Our data are consistent with the existence of CA NTD-NTD interactions and CTD-NTD interactions within the Ty3 VLPs, similar to what is observed in the HIV1 CA 2D crystal structure and in the RSV cryoEM. The fact that these interactions are observed for isolated subdomains and between NTD and CA suggests that they underlie the stability of the mature Ty3 particle.

Finally, this investigation provides further support for our model that Gag3 undergoes conformational switching between unassembled and assembled states [17]. First, isolated NTD interacted preferentially with p27 and CA, compared to Gag3, indicating that the NTD interface may become more accessible during maturation. Second, the CTD failed to interact with Gag3, but interacted readily with the isolated NTD. Third, mutations that blocked native assembly had their most pronounced effect at the level of Gag3, rather than in individual domain interactions, suggesting that exposure of interaction surfaces is not equivalent in Gag3 and intermediate forms. It is also possible that the subdomain interactions play a role in the hypothesized conformational switch: Intramolecular NTD-CTD interactions might help to order assembly by limiting NTD-NTD interactions until precursor localization or RNA association is achieved. Similarly, NC, the presence of which appears to correlate with decreased interaction between Gag3 derivatives and NTD, might help to limit completion of intermolecular interactions until genomic RNA is engaged. Our findings also pose the possibility that two-hybrid assays of retroviral CA subdomains might provide detection of interactions that have thus far been detected in only in crystallographic studies. If successful, such an assay could be useful in high throughput screening for inhibitors of assembly.

## Competing interests

The authors declare that they have no competing interests.

## Authors' contributions

MZ and SBS designed the study and drafted the manuscript. MZ and LZ designed and constructed Ty3 Gag3 mutant collection. MZ and VB subcloned into two-hybrid vectors and together with BI performed the two-hybrid analysis. All authors read and approved the manuscript.

## Supplementary Material

Additional file 1 **Sup. Fig. 1. Interactions of BD M4 mutant Gag3 D60A/R63A with Gag3 wild type, D60A/R63A, G87A, F93A, E148A/K149A, and E190A/R191A**. The D60A/R63A mutation disrupts interactions with wild type and other mutant Gag3 proteins. Images of yeast strain yAH109 containing two-hybrid pGAD or pGBK vectors or expression plasmids as described in text. Cells are shown as representative streaks of independent transformants growing on synthetic dextrose medium lacking tryptophan, leucine, and adenine. Mutants are M4 (D60A/R63A), MHR2 (G87A), MHR4 (F93A), M13 (E148A/K149A), and M18 (E190A/R191A). Binding domain (BD) fusions are labeled in the top row and shown in columns; activation domain (AD) fusions are labeled in the left column and shown in rows. Scoring is based on four individual transformants (two shown). There was variability among individual BD-Gag3/AD-N-terminal domain (NTD) transformants (for example Figure 1, Additional file 7 and Additional file 10). Click here for file

Additional file 2 **Sup. Fig. 2. Interactions of BD MHR2 mutant Gag3 G87A with Gag3 wild type, G87A, F93A, E148A/K149A, and E190A/R191A**. The G87A mutation disrupts interactions with wild type and other mutant Gag3 proteins. Click here for file

Additional file 3 **Sup. Fig. 3. Interactions of BD MHR4 mutant Gag3 F93A with Gag3 wild type, F93A, G87A, E148A/K149A, and E190A/R191A**. The F93A mutation disrupts interactions with wild type and other mutant Gag3 proteins. Click here for file

Additional file 4 **Sup. Fig. 4. Interactions of BD M13 mutant Gag3 E148A/K149A with Gag3 wild type, E148A/K149A, D60A/R63A, G87A, F93A, and E190A/R191A**. The E148A/K149A mutation disrupts interactions with wild type and other mutant Gag3 proteins. Click here for file

Additional file 5 **Sup. Fig. 5. Interactions of BD M18 mutant Gag3 E190A/R191A with Gag3 wild type, E190A/R191A, D60A/R63A, G87A, F93A, and E148A/K149A**. BD E190A/R191A interacts with other mutant Gag3 proteins, although less well with D60A/R63A, G87A, and F93A. Click here for file

Additional file 6 **Sup. Fig. 6. Interactions of BD wild type Gag3 with wild type Gag3, D60A/R63A, G87A, F93A, E148A/K149A, and E190A/R191A**. Wt Gag3 interacts with other mutant Gag3 proteins, although much less well with D60A/R63A, G87A, and F93A. Click here for file

Additional file 7 **Sup. Fig. 7. Interactions of BD capsid (CA) NTD with wild type Gag3, CA NTD, and CA CTD**. BD CA NTD interacts with CA NTD and CA CTD. Click here for file

Additional file 8 **Sup. Fig. 8. BD capsid (CA) NTD interaction with Gag3 wild type and D60A/R63A, G87A, F93A, E148A/K149A, and E190A/R191A**. Mutations in the CA NTD and CA CTD that disrupt interactions in the Gag3 context fail to allow observation of interactions between CA NTD and Gag3. Click here for file

Additional file 9 **Sup. Fig. 9. Interactions between BD capsid (CA) CTD and wild type Gag3, D60A/R63A, G87A, F93A E148A/K149A, and E190A/R191A**. Mutations in Gag3 that disrupt Gag3 interactions in the Gag3 context fail to allow observation of interactions between CA CTD and Gag3. Click here for file

Additional file 10 **Sup. Fig. 10. Interactions between BD Gag3 and wild type Gag3, wild type CA NTD, and capsid (CA) NTD D60A/R63A, CA NTD G87A, and CA NTD F93A**. Mutations in the CA NTD that disrupt interactions in the Gag3 context fail to allow observation of interactions between CA NTD and Gag3. Click here for file

Additional file 11 **Sup. Fig. 11. Interactions between BD capsid (CA) NTD and BD CA CTD with wild type CA NTD, CA NTD D60A/R63A, CA NTD G87A, and CA NTD F93A**. With the exception of D60A/R63A mutations in the CA NTD that disrupt Gag3 interactions do not decrease CA NTD interactions with the CA NTD. D60A/R63A does not decrease interactions with BD CA CTD. Click here for file

Additional file 12 **Sup. Fig. 12. Interactions between BD capsid (CA) CTD M13 mutant E148A/K149A and BD CA CTD M18 mutant E190A/R191A with wild type Gag3, CA NTD, CA NTD D60A/R63A, CA NTD G87A, and CA NTD F93A**. Mutations in CA CTD and CA NTD that disrupt Gag3 interactions do not interfere with observation of interactions between the CA CTD and CA NTD. Click here for file
