# Supplementary figures and images for: Two-hybrid analysis of Ty3 capsid subdomain interactions
Source: Mob DNA. 2010 May 5;1:14. doi: 10.1186/1759-8753-1-14 (PMC2878294; doi:10.1186/1759-8753-1-14)

|        |         |               |
|--------|---------|---------------|
| V/V    | M4/V    | Gag3/<br>Gag3 |
| V/Gag3 | M4/Gag3 |               |
| V/M4   | M4/M4   | M4/MHR2       |
| V/M13  | M4/M13  | M4/MHR4       |
| V/M18  | M4/M18  |               |

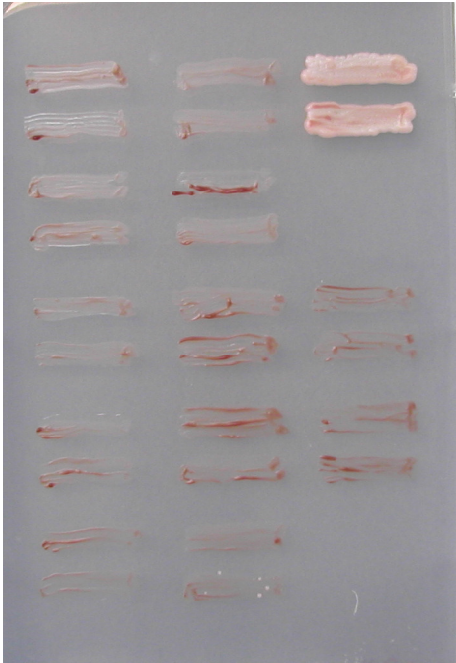

|   |   |     |
|---|---|-----|
| - | - | +++ |
| - | - |     |
| - | - | -   |
| - | - | -   |
| - | - |     |

Fig. 1

Supplement: Additional file 1 — Sup. Fig. 1. Interactions of BD M4 mutant Gag3 D60A/R63A with Gag3 wild type, D60A/R63A, G87A, F93A, E148A/K149A, and E190A/R191A. The D60A/R63A mutation disrupts interactions with wild type and other mutant Gag3 proteins. Images of yeast strain yAH109 containing two-hybrid pGAD or pGBK vectors or expression plasmids as described in text. Cells are shown as representative streaks of independent transformants growing on synthetic dextrose medium lacking tryptophan, leucine, and adenine. Mutants are M4 (D60A/R63A), MHR2 (G87A), MHR4 (F93A), M13 (E148A/K149A), and M18 (E190A/R191A). Binding domain (BD) fusions are labeled in the top row and shown in columns; activation domain (AD) fusions are labeled in the left column and shown in rows. Scoring is based on four individual transformants (two shown). There was variability among individual BD-Gag3/AD-N-terminal domain (NTD) transformants (for example Figure 1, Additional file 7 and Additional file 10). [file 1759-8753-1-14-S1.PDF]

|        |               |               |
|--------|---------------|---------------|
| V/V    | MHR2/V        | Gag3/<br>Gag3 |
| V/Gag3 | MHR2/<br>Gag3 |               |
| V/M4   | MHR2/<br>M4   | MHR2/<br>MHR2 |
| V/M13  | MHR2/<br>M13  | MHR2/<br>MHR4 |
| V/M18  | MHR2/<br>M18  |               |

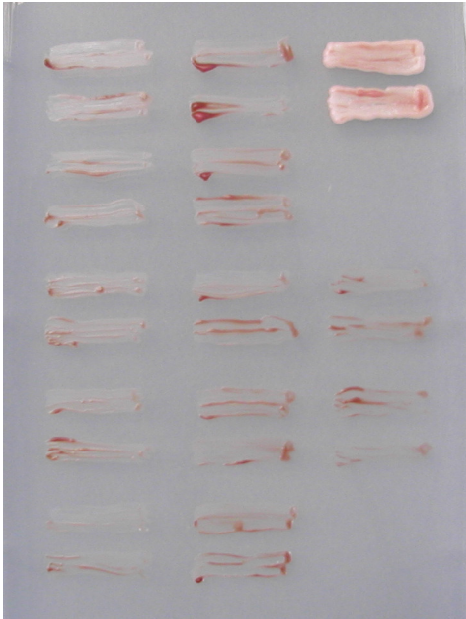

|   |   |     |
|---|---|-----|
| - | - | +++ |
| - | - |     |
| - | - | -   |
| - | - | -   |
| - | - |     |

Fig. 2

Supplement: Additional file 2 — Sup. Fig. 2. Interactions of BD MHR2 mutant Gag3 G87A with Gag3 wild type, G87A, F93A, E148A/K149A, and E190A/R191A. The G87A mutation disrupts interactions with wild type and other mutant Gag3 proteins. [file 1759-8753-1-14-S2.PDF]

|        |               |               |
|--------|---------------|---------------|
| V/V    | MHR4/V        | Gag3/<br>Gag3 |
| V/Gag3 | MHR4/<br>Gag3 |               |
| V/M4   | MHR4/<br>M4   | MHR4/<br>MHR2 |
| V/M13  | MHR4/<br>M13  | MHR4/<br>MHR4 |
| V/M18  | MHR4<br>/M18  |               |

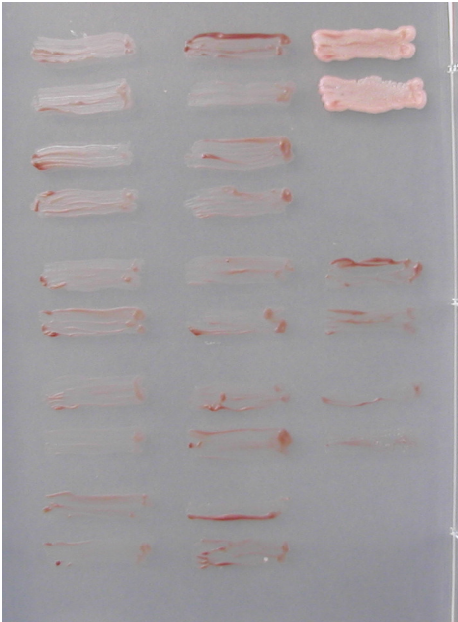

|   |   |     |
|---|---|-----|
| - | - | +++ |
| - | - |     |
| - | - | -   |
| - | - | -   |
| - | - |     |

Fig. 3

Supplement: Additional file 3 — Sup. Fig. 3. Interactions of BD MHR4 mutant Gag3 F93A with Gag3 wild type, F93A, G87A, E148A/K149A, and E190A/R191A. The F93A mutation disrupts interactions with wild type and other mutant Gag3 proteins. [file 1759-8753-1-14-S3.PDF]

|        |              |               |
|--------|--------------|---------------|
| V/V    | M13/V        | Gag3/<br>Gag3 |
| V/Gag3 | M13/<br>Gag3 |               |
| V/M4   | M13/M4       | M13/<br>MHR2  |
| V/M13  | M13/M13      | M13/<br>MHR4  |
| V/M18  | M13/M18      |               |

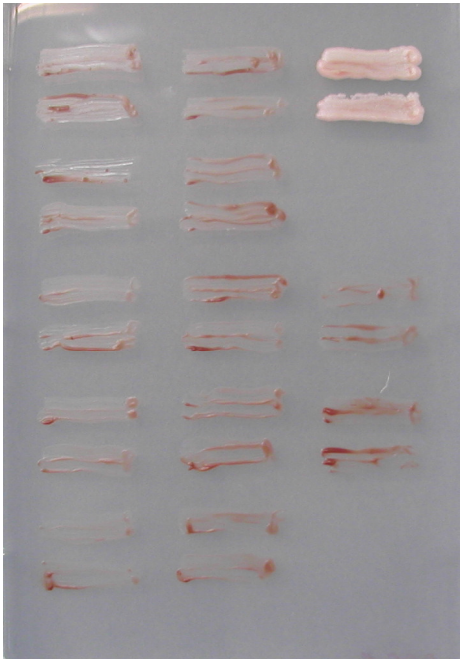

|   |   |     |
|---|---|-----|
| - | - | +++ |
| - | - |     |
| - | - | -   |
| - | - | -   |
| - | - |     |

Fig. 4

Supplement: Additional file 4 — Sup. Fig. 4. Interactions of BD M13 mutant Gag3 E148A/K149A with Gag3 wild type, E148A/K149A, D60A/R63A, G87A, F93A, and E190A/R191A. The E148A/K149A mutation disrupts interactions with wild type and other mutant Gag3 proteins. [file 1759-8753-1-14-S4.PDF]

|        |              |               |
|--------|--------------|---------------|
| V/V    | M18/V        | Gag3/<br>Gag3 |
| V/Gag3 | M18/<br>Gag3 |               |
| V/M4   | M18/<br>M4   | M18/<br>MHR2  |
| V/M13  | M18/<br>M13  | M18/<br>MHR4  |
| V/M18  | M18/<br>M18  |               |

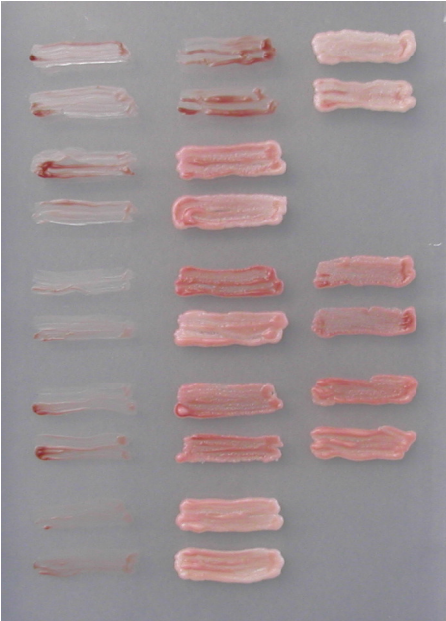

|   |      |      |
|---|------|------|
| - | -    | ++++ |
| - | ++++ |      |
| - | ++   | ++   |
| - | ++   | ++   |
| - | ++++ |      |

Fig. 5

Supplement: Additional file 5 — Sup. Fig. 5. Interactions of BD M18 mutant Gag3 E190A/R191A with Gag3 wild type, E190A/R191A, D60A/R63A, G87A, F93A, and E148A/K149A. BD E190A/R191A interacts with other mutant Gag3 proteins, although less well with D60A/R63A, G87A, and F93A. [file 1759-8753-1-14-S5.PDF]

|        |               |               |
|--------|---------------|---------------|
| V/V    | Gag3/V        |               |
| V/Gag3 | Gag3/<br>Gag3 |               |
| V/M4   | Gag3/<br>M4   | Gag3/<br>MHR2 |
| V/M13  | Gag3/<br>M13  | Gag3/<br>MHR4 |
| V/M18  | Gag3/<br>M18  |               |

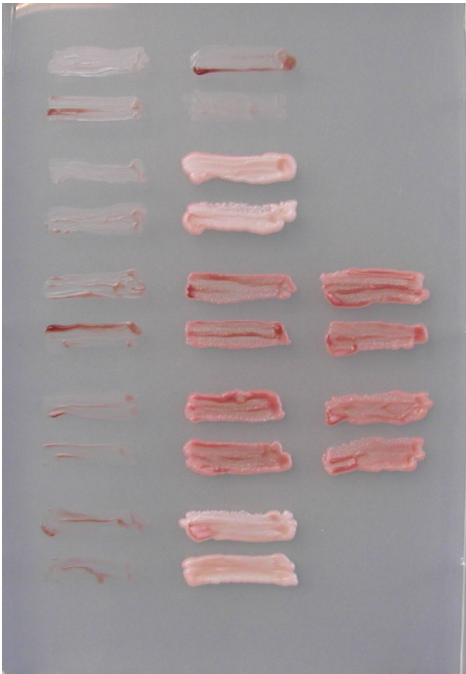

|   |      |    |
|---|------|----|
| - | -    |    |
| - | ++++ |    |
| - | +    | ++ |
| - | ++   | ++ |
| - | ++++ |    |

Fig. 6

Supplement: Additional file 6 — Sup. Fig. 6. Interactions of BD wild type Gag3 with wild type Gag3, D60A/R63A, G87A, F93A, E148A/K149A, and E190A/R191A. Wt Gag3 interacts with other mutant Gag3 proteins, although much less well with D60A/R63A, G87A, and F93A. [file 1759-8753-1-14-S6.PDF]

|        |               |              |              |
|--------|---------------|--------------|--------------|
| V/V    | Gag3/V        | NTD/V        | CTD/V        |
| V/Gag3 | Gag3/<br>Gag3 | NTD/<br>Gag3 | CTD/<br>Gag3 |
|        |               |              |              |
| V/NTD  | Gag3/<br>NTD  | NTD/<br>NTD  | CTD/<br>NTD  |

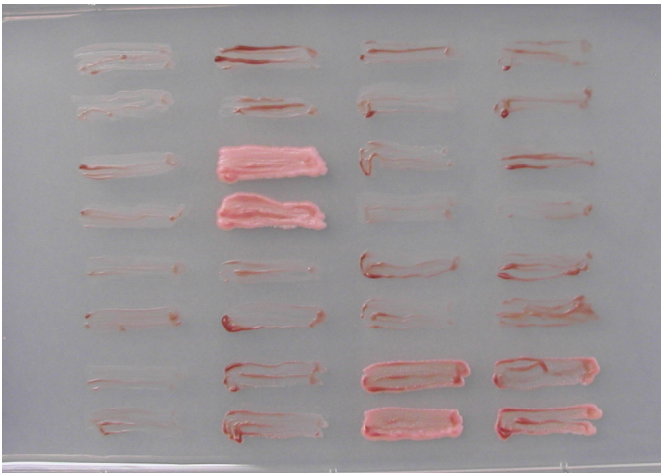

|   |     |    |    |
|---|-----|----|----|
| - | -   | -  | -  |
| - | +++ | -  | -  |
|   |     |    |    |
| - | +/- | ++ | ++ |

Fig. 7

Supplement: Additional file 7 — Sup. Fig. 7. Interactions of BD capsid (CA) NTD with wild type Gag3, CA NTD, and CA CTD. BD CA NTD interacts with CA NTD and CA CTD. [file 1759-8753-1-14-S7.PDF]

|        |              |               |
|--------|--------------|---------------|
| V/V    | NTD/V        | Gag3/<br>Gag3 |
| V/Gag3 | NTD/<br>Gag3 |               |
| V/M4   | NTD/<br>M4   | NTD/<br>MHR2  |
| V/M13  | NTD/<br>M13  | NTD/<br>MHR4  |
| V/M18  | NTD/<br>M18  |               |

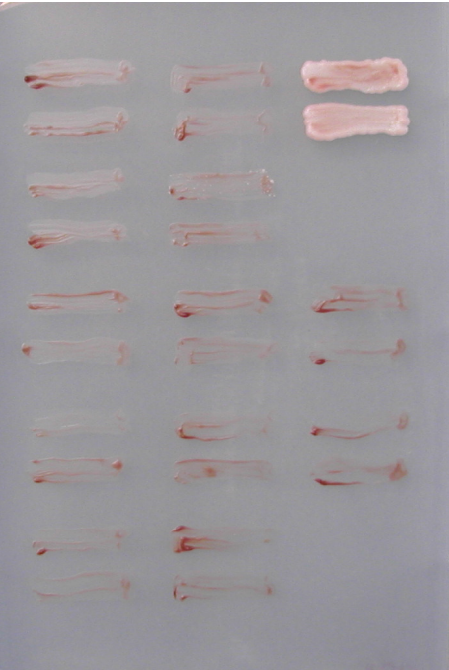

|   |   |      |
|---|---|------|
| - | - | ++++ |
| - | - |      |
| - | - | -    |
| - | - | -    |
| - | - |      |

Fig. 8

Supplement: Additional file 8 — Sup. Fig. 8. BD capsid (CA) NTD interaction with Gag3 wild type and D60A/R63A, G87A, F93A, E148A/K149A, and E190A/R191A. Mutations in the CA NTD and CA CTD that disrupt interactions in the Gag3 context fail to allow observation of interactions between CA NTD and Gag3. [file 1759-8753-1-14-S8.PDF]

|        |              |               |
|--------|--------------|---------------|
| V/V    | CTD/V        | Gag3/<br>Gag3 |
| V/Gag3 | CTD/<br>Gag3 |               |
| V/M4   | CTD/M4       | CTD/<br>MHR2  |
| V/M13  | CTD/M13      | CTD/<br>MHR4  |
| V/M18  | CTD/M18      |               |

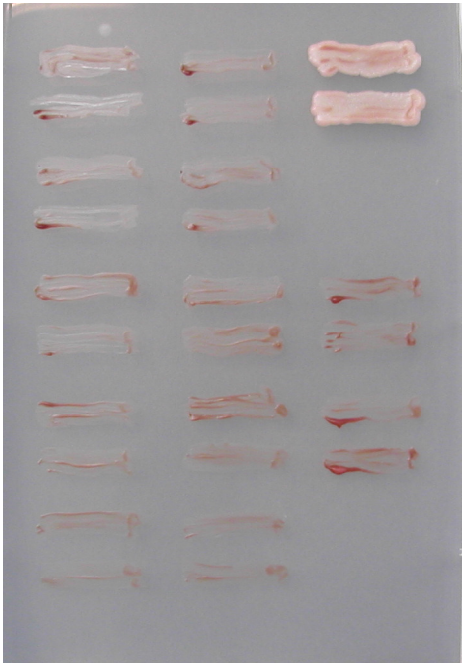

|   |   |     |
|---|---|-----|
| - | - | +++ |
| - | - |     |
| - | - | -   |
| - | - | -   |
| - | - |     |

Fig. 9

Supplement: Additional file 9 — Sup. Fig. 9. Interactions between BD capsid (CA) CTD and wild type Gag3, D60A/R63A, G87A, F93A E148A/K149A, and E190A/R191A. Mutations in Gag3 that disrupt Gag3 interactions in the Gag3 context fail to allow observation of interactions between CA CTD and Gag3. [file 1759-8753-1-14-S9.PDF]
