# Supplementary material for: Two-hybrid analysis of Ty3 capsid subdomain interactions
Source: Mob DNA. 2010 May 5;1:14. doi: 10.1186/1759-8753-1-14 (PMC2878294; doi:10.1186/1759-8753-1-14)
Supplement: Additional file 10 — Sup. Fig. 10. Interactions between BD Gag3 and wild type Gag3, wild type CA NTD, and capsid (CA) NTD D60A/R63A, CA NTD G87A, and CA NTD F93A. Mutations in the CA NTD that disrupt interactions in the Gag3 context fail to allow observation of interactions between CA NTD and Gag3. [file 1759-8753-1-14-S10.PDF]

| | | |
|---|---|---|
| | Gag3/V | V/V |
| V/Gag3 | | Gag3/ Gag3 |
| V/ NTD | Gag3/ NTD | |
| V/ NTDMHR2 | Gag3/ NTDMHR2 | |
| V/ NTDMHR4 | Gag3/ NTDMHR4 | |
| V/ NTDM4 | Gag3/ NTDM4 | |

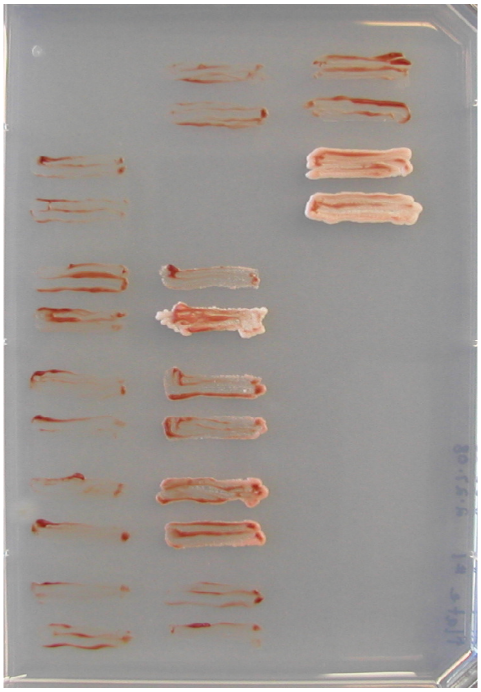

| | | |
|---|---|---|
| | - | - |
| - | | +++ |
| - | +/- | |
| - | +/- | |
| - | +/- | |
| - | - | |

Fig. 10
