# Supplementary material for: Two-hybrid analysis of Ty3 capsid subdomain interactions
Source: Mob DNA. 2010 May 5;1:14. doi: 10.1186/1759-8753-1-14 (PMC2878294; doi:10.1186/1759-8753-1-14)
Supplement: Additional file 11 — Sup. Fig. 11. Interactions between BD capsid (CA) NTD and BD CA CTD with wild type CA NTD, CA NTD D60A/R63A, CA NTD G87A, and CA NTD F93A. With the exception of D60A/R63A mutations in the CA NTD that disrupt Gag3 interactions do not decrease CA NTD interactions with the CA NTD. D60A/R63A does not decrease interactions with BD CA CTD. [file 1759-8753-1-14-S11.PDF]

NTD/V	CTD/V	V/V
NTD/ Gag3	CTD/ Gag3	Gag3/ Gag3
NTD/ NTD	CTD/ NTD	V/ NTD
NTD/ NTDMHR2	CTD/ NTDMHR2	
NTD/ NTDMHR4	CTD/ NTDMHR4	
NTD/ NTDM4	CTD/ NTDM4	

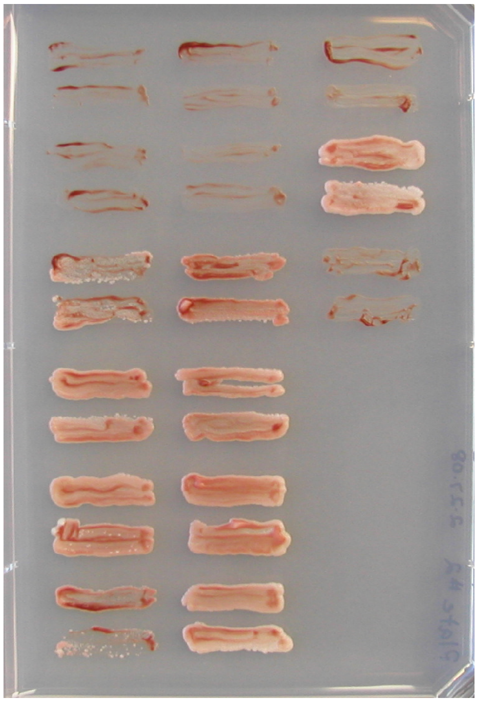

-	-	-
-	-	+++
++	++	-
+++	+++	
+++	+++	
+	+++	

Fig. 11
