# Supplementary material for: Two-hybrid analysis of Ty3 capsid subdomain interactions
Source: Mob DNA. 2010 May 5;1:14. doi: 10.1186/1759-8753-1-14 (PMC2878294; doi:10.1186/1759-8753-1-14)
Supplement: Additional file 12 — Sup. Fig. 12. Interactions between BD capsid (CA) CTD M13 mutant E148A/K149A and BD CA CTD M18 mutant E190A/R191A with wild type Gag3, CA NTD, CA NTD D60A/R63A, CA NTD G87A, and CA NTD F93A. Mutations in CA CTD and CA NTD that disrupt Gag3 interactions do not interfere with observation of interactions between the CA CTD and CA NTD. [file 1759-8753-1-14-S12.PDF]

CTDM13 /V	CTDM18 /V	V/V
CTDM13 /Gag3	CTDM18 /Gag3	Gag3 /Gag3
CTDM13 /NTD	CTDM18 /NTD	CTD /NTD
CTDM13 /NTDMHR2	CTDM18 /NTDMHR2	
CTDM13 /NTDMHR4	CTDM18 /NTDMHR4	
CTDM13 /NTDM4	CTDM18 /NTDM4	

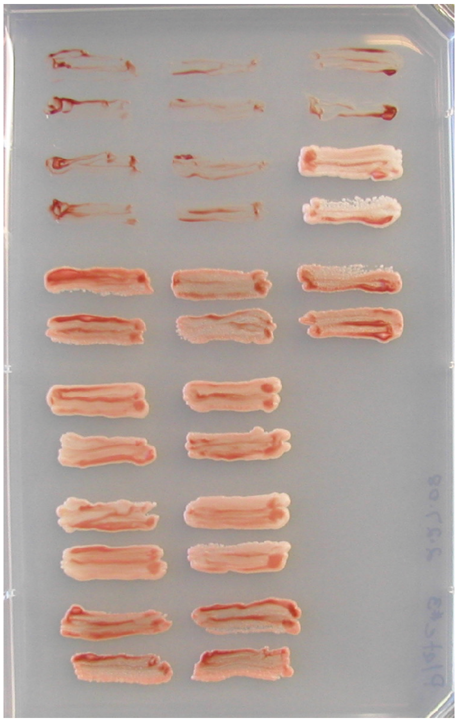

-	-	-
-	-	+++
++	++	++
+++	+++	
+++	+++	
++	++	

Fig. 12
